# Luseogliflozin preserves the pancreatic beta-cell mass and function in *db/db* mice by improving mitochondrial function

**DOI:** 10.1038/s41598-022-13888-6

**Published:** 2022-06-13

**Authors:** Yuki Yamauchi, Akinobu Nakamura, Takashi Yokota, Kiyohiko Takahashi, Shinichiro Kawata, Kazuhisa Tsuchida, Kazuno Omori, Hiroshi Nomoto, Hiraku Kameda, Kyu Yong Cho, Toshihisa Anzai, Shinya Tanaka, Yasuo Terauchi, Hideaki Miyoshi, Tatsuya Atsumi

**Affiliations:** 1 Department of Rheumatology, Endocrinology and Nephrology, Faculty of Medicine and Graduate School of Medicine, Hokkaido University, Sapporo, Japan; 2 Clinical Research and Medical Innovation Center, Hokkaido University Hospital, Sapporo, Japan; 3 Department of Cardiovascular Medicine, Faculty of Medicine and Graduate School of Medicine, Hokkaido University, Sapporo, Japan; 4 Department of Cancer Pathology, Faculty of Medicine, Hokkaido University, Sapporo, Japan; 5 Institute for Chemical Reaction Design and Discovery (WPI-ICReDD), Hokkaido University, Sapporo, Japan; 6 Department of Endocrinology and Metabolism, Graduate School of Medicine, Yokohama City University, Yokohama, Japan; 7 Division of Diabetes and Obesity, Faculty of Medicine and Graduate School of Medicine, Hokkaido University, Sapporo, Japan

**Keywords:** Endocrinology, Medical research

## Abstract

We aimed to determine the mechanism by which the sodium glucose co-transporter 2 inhibitor, luseogliflozin, preserves pancreatic beta-cell mass and function in *db/db* mice. Six-week-old *db/db* mice were fed to standard chow or standard chow containing 0.01% luseogliflozin. After 4 weeks, DNA microarray analysis, real-time PCR analysis, and measurement of mitochondrial respiratory capacity and reactive oxygen species (ROS) generation were performed using isolated islets. Immunohistochemistry and electron microscopic analysis were performed using pancreatic tissues. Metabolites extracted from the islets were measured by capillary electrophoresis mass spectrometry. The expression of genes involved in the tricarboxylic acid (TCA) cycle and electron transport chain was upregulated by luseogliflozin. Luseogliflozin improved the mitochondrial complex II-linked oxidative phosphorylation capacity and reduced ROS generation. Mitochondrial morphology was normally maintained by luseogliflozin. Luseogliflozin increased NK6 homeobox 1 (NKX6.1) expression and TCA cycle metabolites. Relief of glucotoxicity by luseogliflozin may involve lower mitochondrial ROS generation and an improvement in complex II-linked mitochondrial respiration. Reducing ROS generation through preventing complex II damage likely increases NKX6.1 expression and ameliorate glucose metabolism in the TCA cycle, contributing to the protection of pancreatic beta-cells. Protection of complex II in pancreatic beta-cells represents a novel therapeutic target for type 2 diabetes.

## Introduction

Type 2 diabetes is characterized by relative insulin deficiency and insulin resistance in target organs^1^. Insulin resistance is mainly caused by overnutrition and obesity, which results in decreasing the metabolic response to insulin in the liver, skeletal muscle, and adipose tissue^2^. In contrast, insulin deficiency is associated with changes in pancreatic beta-cell mass and function^3^. Pancreatic beta-cell mass in patients with type 2 diabetes is lower than that in individuals without type 2 diabetes, regardless of their body mass index^4,5^. In addition, impaired pancreatic beta-cell function is evident during the early stages of type 2 diabetes, and the beta-cell impairment extends further according to the duration of diabetes^6,7^. Therefore, the maintenance of beta-cell mass and function is one of the most principle therapeutic strategies for patients with type 2 diabetes.

Sodium glucose co-transporter 2 (SGLT2) inhibitors downregulate the reabsorption of glucose from the proximal renal tubules which results in a reduction in plasma glucose concentration^8^. Because SGLT2 is not expressed in pancreatic beta-cells, SGLT2 inhibitors have indirect effects on pancreatic beta-cells via the lower circulating glucose concentration^9^. Among SGLT2 inhibitors, luseogliflozin is a highly selective SGLT2 inhibitor^10^. Our previous study showed that luseogliflozin treatment for 4 weeks reduced the glucose concentration in *db/db* mice. Furthermore, luseogliflozin increased the pancreatic beta-cell mass and proliferation capacity, the insulin-to-glucose ratio, and the insulin content of the pancreatic islets^11^. However, the underlying mechanisms of the protective effects of luseogliflozin on pancreatic beta-cell mass and function in type 2 diabetes remain to be determined.

The mitochondria play a key role in the maintenance of pancreatic beta-cell function^12^. Mitochondrial oxidative metabolism in the pancreatic beta-cells was low in mice with diabetes^13^. Furthermore, the mitochondria are a major source of reactive oxygen species (ROS), and because of their proximity, mitochondria represent a primary target of the ROS they generate. Hyperglycemia induces excess mitochondrial ROS generation in pancreatic beta-cells^14^. Taken together, mitochondrial dysfunction in pancreatic beta-cells may contribute to the development of type 2 diabetes.

To investigate the underlying mechanism of the beneficial effects of luseogliflozin in the pancreatic beta-cells, we aimed to determine whether luseogliflozin (1) regulates mitochondrial respiratory capacity and reduces mitochondrial ROS generation in pancreatic islets; (2) prevents islet mitochondrial structural damage; (3) increases the expression of NK6 homeobox 1 (NKX6.1), a key player in pancreatic beta-cell proliferation and maturation; and (4) improves the metabolic pattern in pancreatic islets; in *db/db* mice.

## Results

### Luseogliflozin upregulated the expression of genes involved in glucose uptake, the tricarboxylic acid (TCA) cycle, and the electron transport chain (ETC)

**Figure 1:**
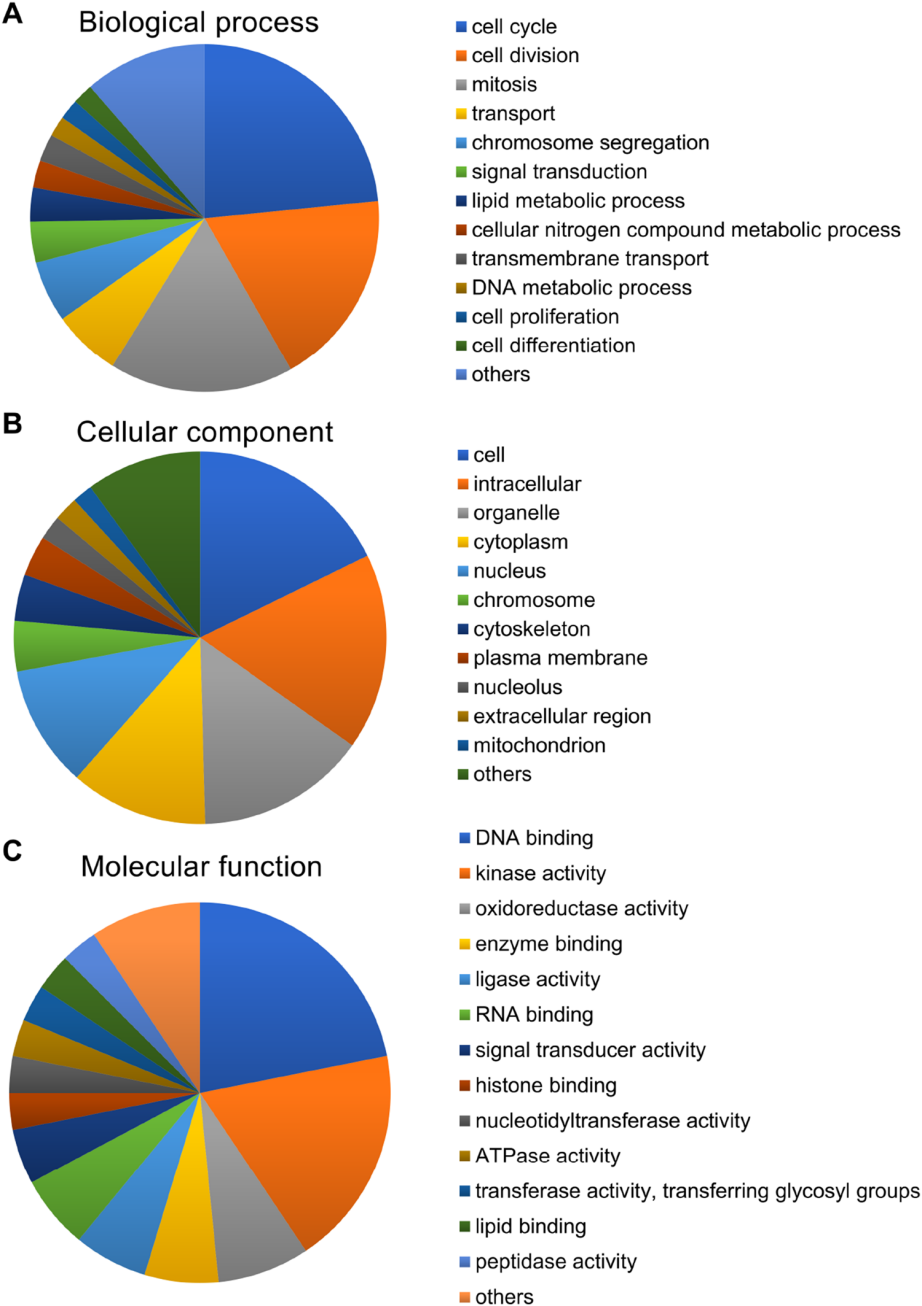
Gene ontology analysis of the genes that were upregulated in the pancreatic islets of 10-week-old *db/db* mice treated with luseogliflozin. Upregulated genes in the luseo group in the microarray analysis were functionally categorized based on gene ontology annotation. (**A**) Biological process. (**B**) Cellular component. (**C**) Molecular function.

To identify genes that play a role in the pancreatic beta-cell protection, we compared the gene expression in isolated islets from mice of the two groups by microarray analysis. This showed that 473 genes were differentially expressed (164 upregulated and 309 downregulated in the luseo group). Cytochrome c oxidase subunit 6A2 (*Cox6a2*), one of the ETC complex IV subunits, and solute carrier family 2 member 2 (*Slc2a2*), also known as glucose transporter 2 (*Glut2*), were identified among the upregulated genes in the luseo group (Table 1). Genes associated with pancreatic beta-cell mass and function were not included among the top 10 most downregulated genes in the luseo group (Table 1). Pathway analysis showed that four pathways were significantly upregulated and six significantly downregulated (Table 2). Some of the upregulated pathways such as fatty acid biosynthesis and glycolysis and gluconeogenesis involve pyruvate carboxylase (*Pcx*), which is associated with glucose metabolism in the TCA cycle. Gene ontology (GO) biological process analysis for the upregulated genes in the luseo group showed that these genes were involved in the cell cycle, cell division, and mitosis. (Fig. 1A). These upregulated genes were mainly concentrated in the cell, intracellular region, and organelles according to the GO cellular component analysis (Fig. 1B) and functioned in areas such as DNA binding, kinase activity, and oxidoreductase activity according to the GO molecular function analysis (Fig. 1C). In particular, the results of the GO biological process analysis are consistent with our previous findings that luseogliflozin increases the pancreatic beta-cell proliferation capacity in *db/db* mice^11^. Taken together, the amelioration of pancreatic beta-cell dysfunction as well as lower proliferation capacity induced by luseogliflozin in *db/db* mice appears to link to the upregulation of glucose uptake, the mitochondrial TCA cycle, and the ETC in the pancreatic beta-cells.

**Table 1:** Top 10 up- and downregulated genes in the luseogliflozin-treated group.

Upregulated	Fold-change	*P*-value	Downregulated	Fold-change	*P*-value
Gene symbol			Gene symbol		
Fam151a	5.20	0.02	Spink3	0.09	< 0.01
Cox6a2	4.33	0.01	Tm4sf20	0.11	< 0.01
Slc2a2	3.98	< 0.01	Cck	0.13	0.02
Top2a	3.63	< 0.01	Pla2g1b	0.16	< 0.01
Casc5	3.43	< 0.01	Prss1	0.20	< 0.01
Angptl7	3.23	< 0.01	Gp2	0.21	0.03
Mki67	3.23	0.03	Pnliprp2	0.22	< 0.01
Pbk	3.08	0.04	Pdia2	0.22	0.01
Ccna2	2.88	0.03	Cuzd1	0.22	0.02
Maob	2.82	< 0.01	Serpini2	0.23	0.02

*P* values were determined using Student’s *t* test.

**Table 2:** Up- and downregulated pathways in the luseogliflozin-treated group.

	Changed genes	Total genes	Z-score	*P*-value	Gene symbol
**Upregulated pathways**					
Fatty acid biosynthesis WP336_71737	2	22	4.91	0.01	Pcx, Hadh
IL7 signaling pathway WP297_69128	2	44	3.20	0.04	Ccna2, Ccnd2
Tryptophan metabolism WP79_73389	2	44	3.20	0.04	Hadh, Maob
Glycolysis and gluconeogenesis WP157_69361	2	47	3.06	0.04	Pcx, Slc2a2
**Downregulated pathways**					
Dopaminergic neurogenesis WP1498_60839	3	29	3.72	0.01	Aldh1a1, Shh, Th
Endochondral ossification WP1270_72216	4	62	3.01	0.02	Enpp1, Ctsl, Adamts5, Plat
Glucocorticoid and mineralocorticoid metabolism WP495_71740	2	13	3.93	0.02	Hsd11b1, Cyp17a1
Retinol metabolism WP1259_71742	3	38	3.06	0.03	Aldh1a, Cd36, Bcmo1
Biogenic amine synthesis WP522_69135	2	15	3.59	0.03	Ache, Th
Blood clotting cascade WP460_71727	2	20	2.97	0.04	Vwf, Plat

*P* values were determined using two-tailed Fisher’s exact tests.

**Figure 2:**
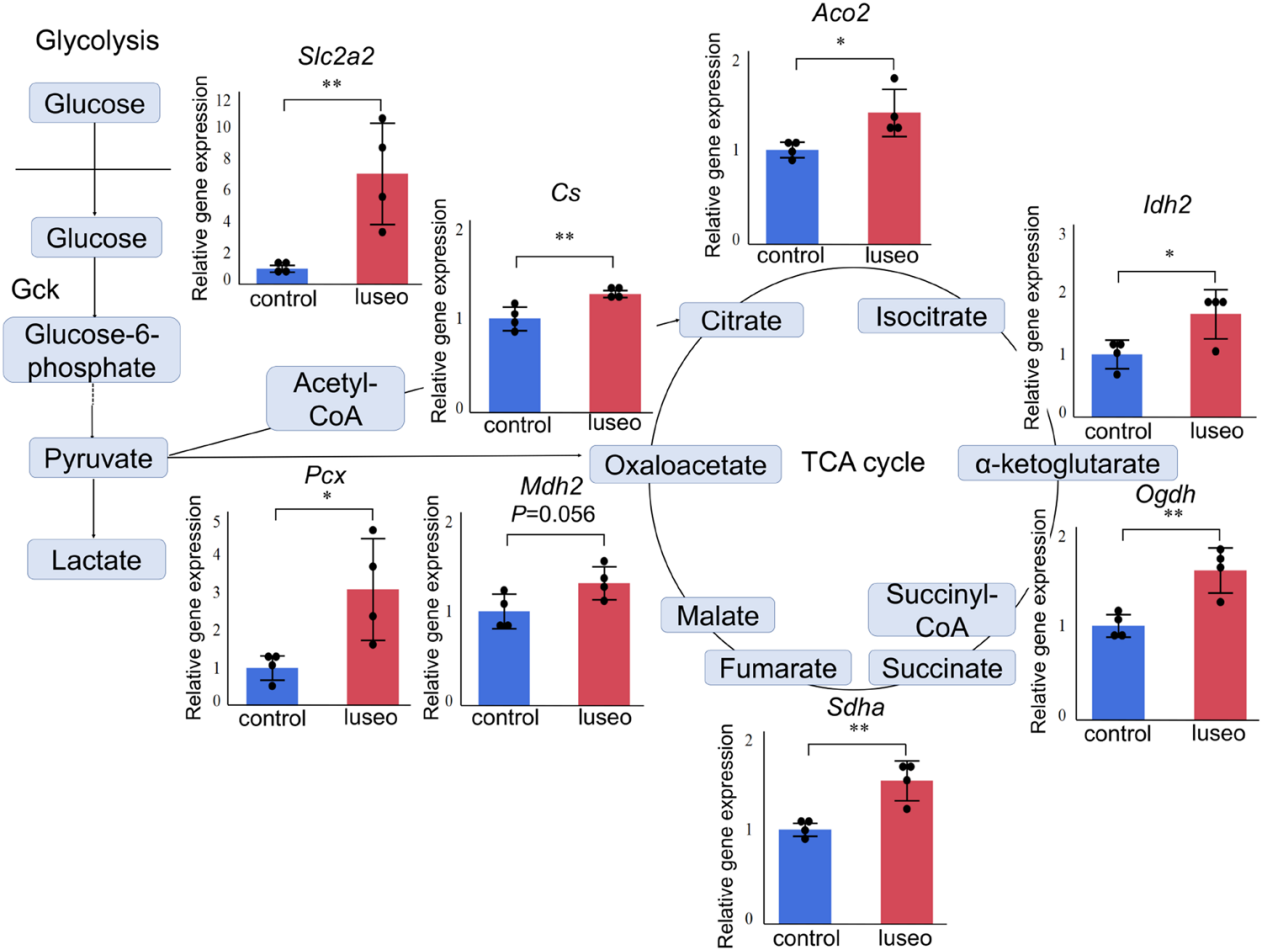
Effects of luseogliflozin on the expression of key genes in the pancreatic islets of 10-week-old *db/db* mice. Gene expression of *Slc2a2, Pcx, Cs, Aco2, Idh2, Ogdh, Sdha,* and *Mdh2* in the control and luseo groups, measured using real-time PCR (*n* = 4). The data were normalized to *Gapdh* expression. Values are mean ± SD. *P* values were determined using Student’s *t* test. *P < 0.05; **P < 0.01. The figure was created by Microsoft PowerPoint® 2016 (https://www.microsoft.com/microsoft-365/powerpoint). *Slc2a2*, solute carrier family 2 member 2; *Pcx*, pyruvate carboxylase; *Cs*, citrate synthase; *Aco2*, aconitase 2; *Idh2*, isocitrate dehydrogenase 2; *Ogdh*, oxoglutarate dehydrogenase; *Sdha*, succinate dehydrogenase complex flavoprotein subunit a; *Mdh2*, malate dehydrogenase 2, *Gapdh*, glyceraldehyde 3-phosphate dehydrogenase.

To confirm the changes in gene expression identified in the microarray analysis, we performed real-time PCR analysis, showing that luseogliflozin significantly upregulated the expression of *Slc2a2* and *Pcx* in the pancreatic islets (Fig. 2). The expression of genes encoding proteins involved in the TCA cycle or the ETC was also significantly upregulated in the luseo group (Fig. 2 and Supplementary Fig. S1A).

### Luseogliflozin improved mitochondrial respiratory capacity in complex II and reduced mitochondrial ROS generation

**Figure 3:**
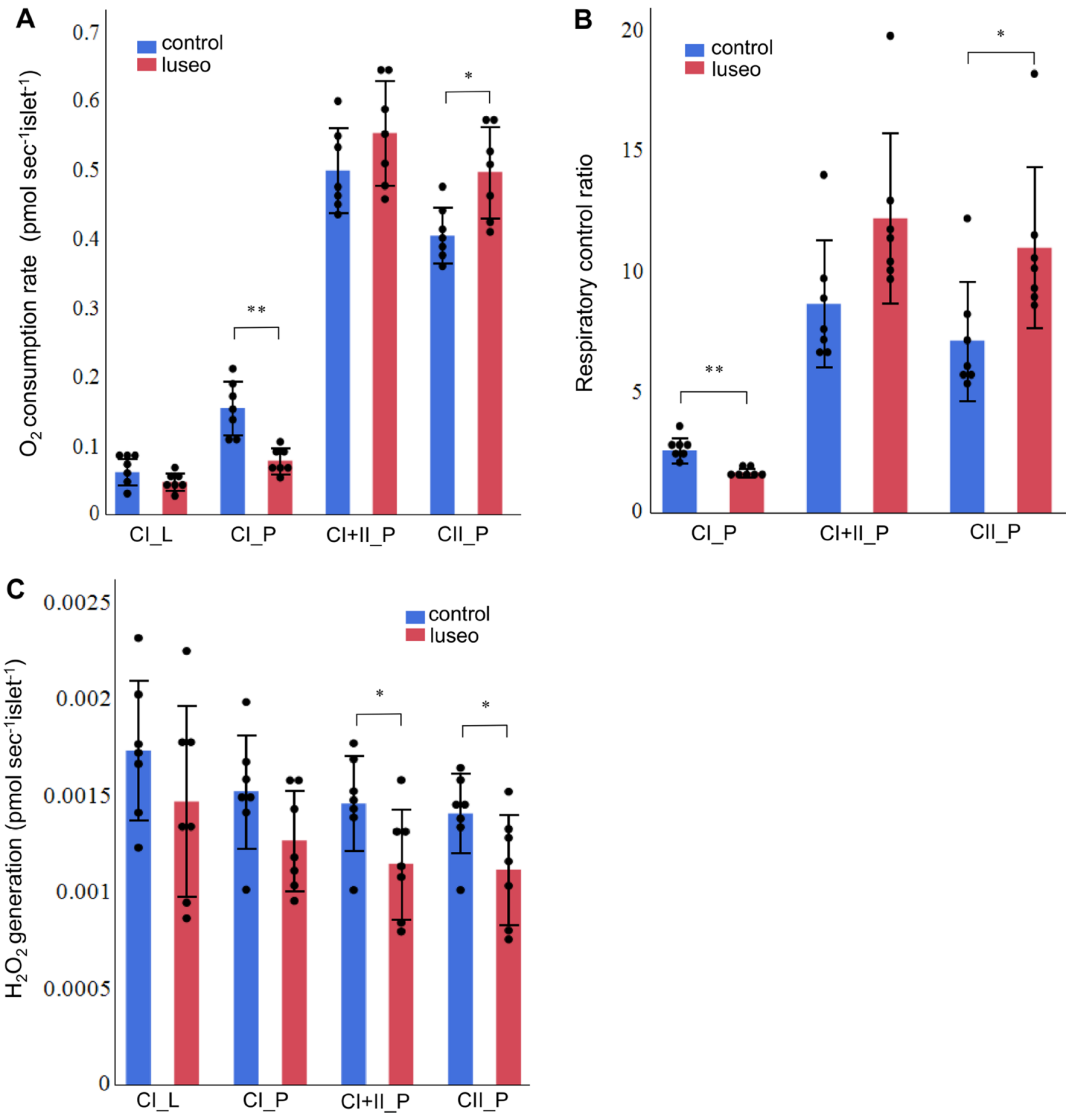
Effects of luseogliflozin on the mitochondrial respiratory capacity and mitochondrial ROS generation of the pancreatic islets of 10-week-old *db/db* mice. (**A**) Mitochondrial respiratory capacity during each state in pancreatic islets from the control and luseo groups (*n* = 7). (**B**) Respiratory control ratio with each complex substrate in pancreatic islets from the control and luseo groups (*n* = 7). (**C)** Mitochondrial H_2_O_2_ generation during each state in pancreatic islets from the control and luseo groups (*n* = 7). Values are mean ± SD. *P* values were determined using Student’s *t* test. *P < 0.05; **P < 0.01. CI, complex I-linked substrates; CI + II, complex I + II-linked substrates; CII, complex II-linked substrates; *L*, leak state; *P*, oxidative phosphorylation.

The luseo group had a higher islet complex II-linked OXPHOS capacity (CII_P) than the control group (Fig. 3A). Although the islet complex I-linked OXPHOS capacity (CI_P) was lower in the luseo group (Fig. 3A), the substrate control ratio (SCR) of complex II was markedly higher than that of complex I in the pancreatic islets of *db/db* mice (Supplementary Fig. S2A), implying that the mitochondria in pancreatic islets are reliant on complex II-linked OXPHOS. The RCR, a parameter that reflects mitochondrial respiratory capacity independently of the number of islets and their mitochondrial density, with complex II-linked substrates was significantly higher in the luseo group (Fig. 3B), which is indicative of greater intrinsic mitochondrial respiration in the islets of *db/db* mice after luseogliflozin treatment. However, no significant difference in complex IV capacity was found between the groups (Supplementary Fig. S2B).

The luseo group showed lower mitochondrial H_2_O_2_ generation during state 3 with either complex II-linked or complex I + II-linked substrates than the control group (Fig. 3C). However, no significant differences were found in the mitochondrial H_2_O_2_ generation during states 2 and 3 with complex I-linked substrates between the two groups (Fig. 3C).

### Luseogliflozin improved the mitochondrial network and prevented mitochondrial structural damage

**Figure 4:**
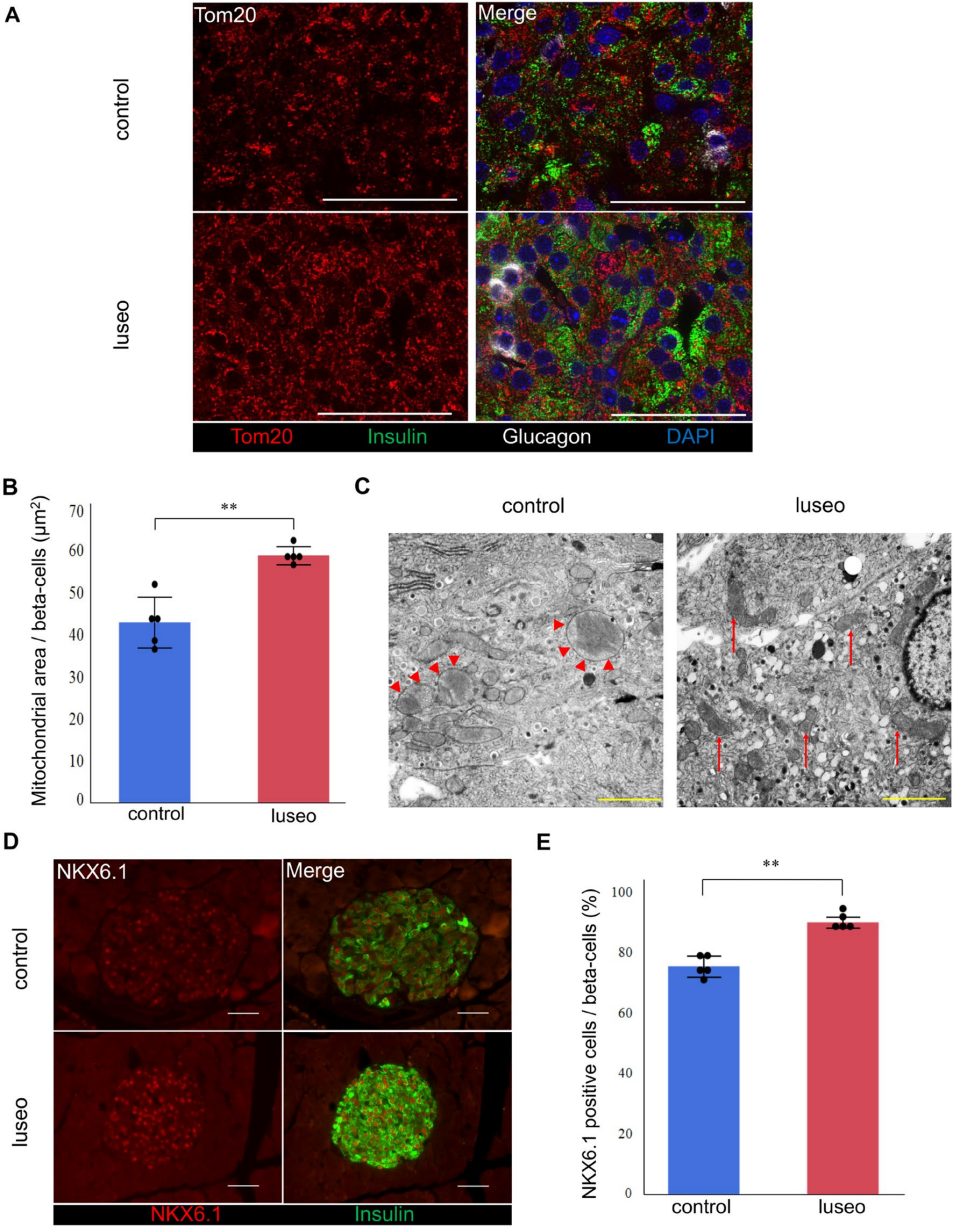
Effects of luseogliflozin on the mitochondrial morphology and NKX6.1 expression in the pancreatic beta-cells of 10-week-old *db/db* mice. (**A**) Images of pancreatic beta-cells from the control and luseo groups stained with Tom 20 (mitochondria, red), insulin (green), glucagon (white), and DAPI (nucleus, blue). Scale bars: 50 µm. (**B**) Mitochondrial area in the pancreatic beta-cells (*n* = 5). (**C**) Electron microscopic analysis of pancreatic beta-cells. Red arrowhead: swelling of mitochondria, red arrows: normal mitochondria. Scale bars: 2 µm. (**D**) Images of pancreatic beta-cells from the control and luseo groups, stained for NKX6.1 (red) and insulin (green). Scale bars: 50 µm. (**E**) Ratio of the number of NKX6.1 and insulin double-positive (co-staining) cells to the total number of insulin positive (beta) cells (%) (*n* = 5). Values are mean ± SD. *P* values were determined using Student’s *t* test. **P < 0.01.

We next evaluated the mitochondrial morphology. We performed insulin and glucagon staining to identify the pancreatic alpha- and beta-cells, and pancreatic beta-cells were analyzed in detail. According to the Tom20 staining, the mitochondrial network was fragmented in the control group but was extended throughout the pancreatic beta-cells in the luseo group (Fig. 4A). Consistently, the mitochondrial area in the luseo group was significantly larger than that in the control group (Fig. 4B). Furthermore, electron microscopic analysis revealed that mitochondrial swelling and disintegration of cristae were more frequently observed in the control group. In contrast, the morphological aspects of mitochondria were well preserved in the luseo group (Fig. 4C).

### Luseogliflozin increased the expression of NKX6.1

It has been reported that a reduction in oxidative stress restores the expression of NKX6.1, which is selectively downregulated in *db/db* mice islets^15^. NKX6.1 directly regulates the transcription of *Glut2* and *Pcx* and contributes to pancreatic beta-cell proliferation by regulating the expression of cyclin D2 (*Ccnd2*)^16^. Therefore, we next quantitated the expression of these two genes. The expression of *Nkx6.1* and *Ccnd2* was significantly higher in the luseo group than in the control group (Supplementary Fig. S1B), being consistent with our previous findings^11^. Furthermore, immunohistochemical analysis showed that the proportion of NKX6.1 positive beta-cells was significantly higher in the luseo group than in the control group (Fig. 4D, E). These results suggest that the reduction in mitochondrial ROS generation induced by luseogliflozin results in an increase in the expression of NKX6.1, and therefore that of *Glut2*, *Pcx*, and *Ccnd2*, which leads to pancreatic beta-cell proliferation in *db/db* mice.

### Luseogliflozin induced a shift from predominant glycolysis to predominant TCA cycle, and increased the concentrations of metabolites involved in insulin secretion

**Figure 5:**
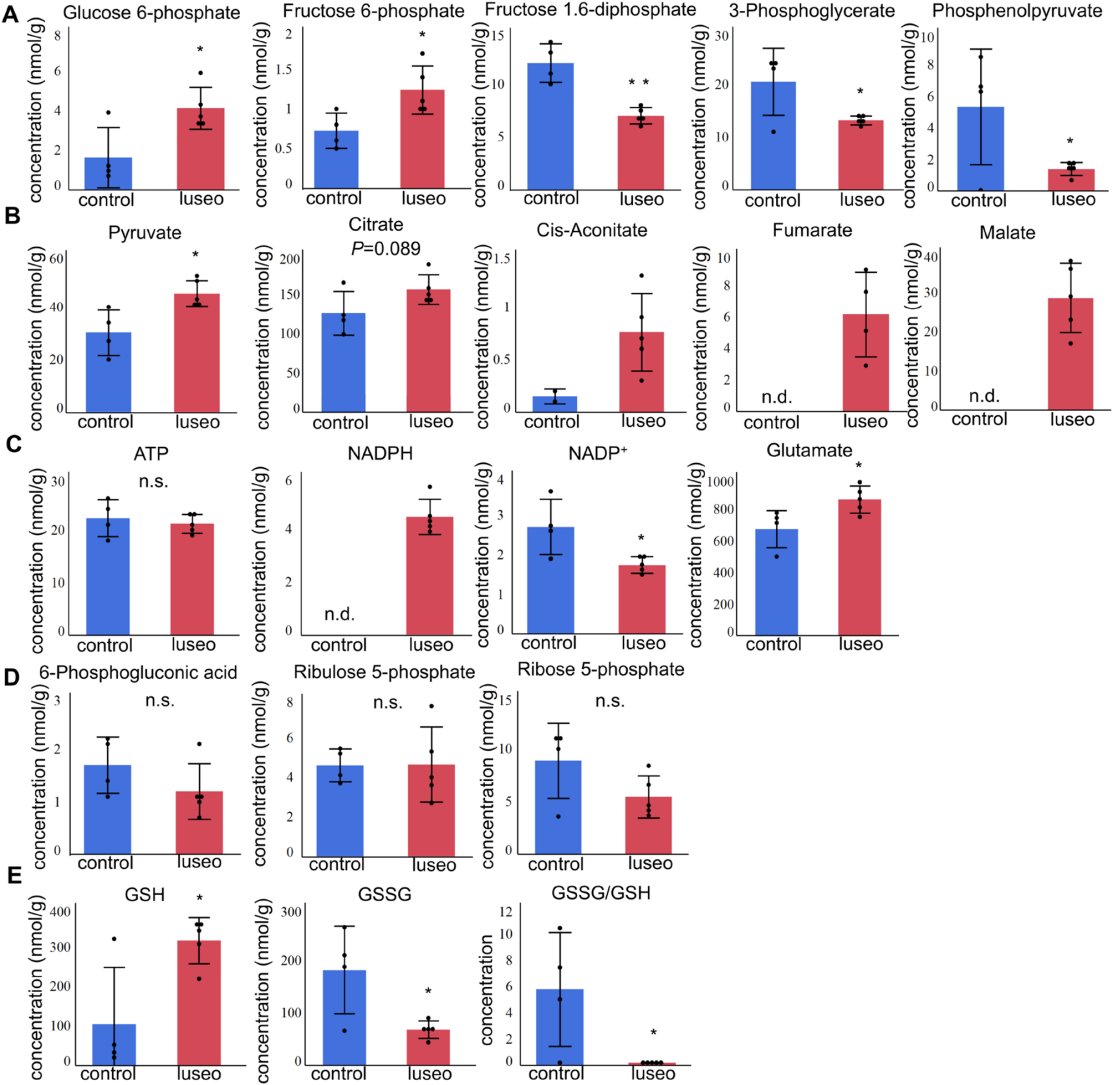
Effects of luseogliflozin on the metabolite concentrations in the pancreatic islets of 10-week-old *db/db* mice. (**A**) Metabolites involved in glycolysis in pancreatic islets from the control (*n* = 4) and luseo (*n* = 5) groups. (**B**) Metabolites involved in the TCA cycle in pancreatic islets from the control (*n* = 4) and luseo (*n* = 5) groups. **(C**) Concentrations of ATP, NADPH, NADP^+^, and glutamate in pancreatic islets from the control (*n* = 4) and luseo (*n* = 5) groups. (**D)** Metabolites involved in the pentose phosphate pathway in pancreatic islets from the control (*n* = 4) and luseo (*n* = 5) groups. **(E)** Metabolites involved in glutathione metabolism in pancreatic islets from the control (*n* = 4) and luseo (*n* = 5) groups. Values are mean ± SD. *P* values were determined using Student’s *t* test. *P < 0.05; **P < 0.01. n.d., not detected; n.s.; not significant, GSH; reduced glutathione, GSSG; oxidized glutathione.

To characterize the glucose metabolism occurring through glycolysis and the TCA cycle, we performed a metabolomic analysis of pancreatic islets. The concentrations of glucose 6-phosphate and fructose 6-phosphate were significantly higher in the luseo group (Fig. 5A), reflecting greater glucose uptake in the luseo group. In contrast, the concentrations of fructose 1,6-diphosphate, 3-phosphoglyerate, and phosphoenolpyruvate in the luseo group were significantly lower than those in the control group (Fig. 5A). Furthermore, the concentrations of the metabolites related to the TCA cycle, including pyruvate, citrate, cis-aconitate, fumarate, and malate, were all higher in the luseo group than in the control group (Fig. 5B). These results showed that luseogliflozin shifts metabolism from a predominantly glycolytic pattern to TCA cycle predominates. This metabolic shift was consistent with the upregulation of genes involved in the TCA cycle and the improved mitochondrial function.

Whereas no significant difference in the ATP concentration was observed between the two groups, the concentrations of NADPH and glutamate, which are metabolites derived from the TCA cycle and associated with insulin secretion^17^, were higher in the luseo group than in the control group (Fig. 5C). Although the pentose phosphate pathway (PPP) is associated with NADPH synthesis and cell proliferation^18^, no significant differences in the concentrations of metabolites in the PPP were identified (Fig. 5D). Finally, the concentration of reduced glutathione (GSH) was significantly increased by luseogliflozin, while that of oxidized glutathione (GSSG) was significantly reduced (Fig. 5E). As a result, the GSSG/GSH ratio, an indicator of cellular oxidative stress, was reduced by luseogliflozin. These results suggest that luseogliflozin increases the concentrations of important TCA cycle metabolites. Those effects of luseogliflozin may further contribute to its protective effect in pancreatic beta-cells.

## Discussion

To the best of our knowledge, this is the first study to show that the protective effect of an SGLT2 inhibitor in pancreatic beta-cells occurs via an improvement in mitochondrial function. We have demonstrated that luseogliflozin improves islet complex II-linked mitochondrial respiratory capacity and reduces mitochondrial ROS generation in *db/db* mice. Furthermore, luseogliflozin maintained normal mitochondrial morphology and increased the expression of NKX6.1, presumably due to the reduction in ROS generation. The amelioration of the mitochondrial dysfunction and the increase in NKX6.1 expression induced by luseogliflozin resulted in a shift from predominantly glycolytic metabolism toward TCA cycle dominance, further contributing to its protective effect in pancreatic beta-cells.

Our results appeared to be mainly due to the relief of glucotoxicity by luseogliflozin because SGLT2 inhibitors do not act directly on pancreatic beta-cells^9^. Indeed, we confirmed that luseogliflozin treatment did not affect *Nkx6.1* expression in a pancreatic beta-cell line (see Supplementary Fig. S3). In addition, previous reports revealed that hyperglycemia alone was sufficient to impair glucose metabolism in mitochondria, resulting in pancreatic beta-cell dysfunction^13^. Taken together, our results could be explained by the hypoglycemic effect of luseogliflozin.

Chronic hyperglycemia causes glucotoxicity in pancreatic beta-cells in part due to an increase in ROS generation^19,20^. Excess mitochondrial ROS generation creates a vicious cycle of mitochondrial dysfunction and further ROS generation^21^, leading to the pancreatic beta-cell dysfunction. Here, we have shown that luseogliflozin ameliorates hyperglycemia and reduces excess mitochondrial ROS generation in pancreatic beta-cells. In addition, the protein expression of mitochondrial complexes was preserved by luseogliflozin (see Supplementary Fig. S4). Thus, the relief of glucotoxicity by luseogliflozin may prevent pancreatic beta-cell damage by breaking this vicious cycle. Furthermore, we have shown that the GSH content of pancreatic islets was increased by luseogliflozin. Pancreatic beta-cells are susceptible to be exposed by oxidative stress because the expression levels of antioxidant enzymes in islets are very low^22^. Our results suggest that luseogliflozin might protect pancreatic beta-cells by increasing antioxidants, such as GSH, as well as by reducing oxidative stress.

No previous detailed studies have examined the effects of SGLT2 inhibitors on mitochondrial respiratory capacity in pancreatic islets. Here, we have demonstrated that complex II-linked OXPHOS predominates over complex I-linked OXPHOS in pancreatic islets, as shown by a higher SCR for complex II than for complex I. Furthermore, luseogliflozin increased mitochondrial OXPHOS capacity and there was less mitochondrial ROS generation in the presence of complex II-linked substrates. Although complex I and III are also significant sources of ROS^23^, complex II has been recently recognized as one of the most potent sources of ROS^24,25^. Thus, mitochondrial ETC complex II may play a key role in the maintenance of normal mitochondrial function in pancreatic beta-cells.

We have also shown that luseogliflozin improves the mitochondrial network. Mitochondria form a network through fusion, fission, and mitophagy, and an intact network is important for their function^26^. Pancreatic beta-cells exposed to environments rich in nutrients, such as hyperglycemia, tend to show fragmentation of their mitochondrial network and lose the ability to undergo fusion, resulting in beta-cell apoptosis^27^. We demonstrated that luseogliflozin increased mitochondrial fusion (see Supplementary Fig. S5), suggesting that luseogliflozin maintained the mitochondrial network by improving the balance of mitochondrial fusion and fission. Additionally, swollen mitochondria represent greater oxidative stress, leading to impaired pancreatic beta-cell function^28,29^. We showed that luseogliflozin prevents mitochondrial swelling. Thus, luseogliflozin may prevent mitochondrial damage in pancreatic beta-cells by reducing mitochondrial ROS generation.

In our previous study, we found that luseogliflozin preserves pancreatic beta-cell mass and function by increasing the expression of transcription factors, such as V-maf musculoaponeurotic fibrosarcoma oncogene homolog A (*Mafa*) and Pancreas/duodenum homeobox 1 (*Pdx1*), via a suppression of ROS generation^11^. In the present study, we have shown that luseogliflozin increases the expression of NKX6.1, a key mediator of pancreatic beta-cell proliferation and maturation. Because the expression of NKX6.1 is restored by a reduction in oxidative stress^15^, the reduction in mitochondrial ROS generation induced by luseogliflozin may cause the increase in NKX6.1 expression. NKX6.1 contributes to beta-cell proliferation through an increase in the expression of *Ccnd2*^16^. Also, NKX6.1 directly regulates the transcription of *Pcx*, amplifying anaplerosis and providing substrates for beta-cell proliferation, such as NADPH and malate, which are generated by the TCA cycle^30,31^. In the present study, the expression of *Ccnd2* and *Pcx* was upregulated and the islet concentrations of NADPH and malate were increased by luseogliflozin. Therefore, the increase in NKX6.1 expression induced by luseogliflozin may have a significant impact on beta-cell proliferation.

Luseogliflozin increased the concentration of NADPH, which is derived from the TCA cycle and is one of the key metabolites in insulin secretion as well as pancreatic beta-cell proliferation^17^. Previous studies suggested that NADPH inhibited pancreatic beta-cell repolarization by inactivating voltage-gated potassium channels, and increases calcium influx triggering the exocytosis of insulin granules^32,33^. Furthermore, we have shown that the concentration of glutamate is increased by luseogliflozin. Glutamate is generated from a TCA cycle intermediate and is considered to be a second insulin secretion amplifier^34^. Additionally, a recent study demonstrated that glutamate stimulated incretin-induced insulin secretion^35^. Taken together, these findings suggest that NADPH and glutamate derived from the TCA cycle may play an important role in the maintenance of insulin secretion by luseogliflozin.

Some existing antidiabetic agents are reported to have effects on mitochondrial ETC. Metformin specifically inhibits complex I and suppresses gluconeogenesis in the liver through the activation of AMP-activated protein kinase^36,37^. Imeglimin, a novel oral antidiabetic agent, restores complex III respiratory capacity while partially inhibiting complex I in the liver, which results in a decrease in excess ROS generation from complex I^38,39^. From the therapeutic viewpoint, our findings suggest that the maintenance of the complex II-linked OXPHOS capacity and the reduction in ROS generation from complex II contribute to the protective effect of luseogliflozin in pancreatic beta-cells. Although the problem of tissue selectivity remains to be addressed in the future, the protection of complex II in pancreatic beta-cells may represent a novel therapeutic target for type 2 diabetes.

The present study had several limitations. First, we did not evaluate the effects of luseogliflozin on middle-aged *db/db* mice (late phase of diabetes). Also, the long-term effects of luseogliflozin were not examined. We performed this study in six-week-old *db/db* mice (early phase of diabetes) because the earlier luseogliflozin treatment was started, the greater the protective effects of luseogliflozin on pancreatic beta-cells became^11,40^. However, the effects of luseogliflozin at different ages and term with the same analysis should be performed in the near future. Second, we could not compare the effects of luseogliflozin with those of other antidiabetic agents because they act directly on pancreatic beta-cells or do not lower the glucose concentration to the same extent in vivo. Although insulin treatment reduces the glucose concentration, an effect of insulin signaling on pancreatic beta-cells cannot be excluded^41^. Therefore, determining whether luseogliflozin treatment have specific effects on pancreatic beta-cells as well as relieving hyperglycemia in vivo has been difficult and that remains a subject for future investigation.

In conclusion, the relief of glucotoxicity by luseogliflozin is associated with a reduction in mitochondrial ROS generation and an improvement in complex II-linked mitochondrial OXPHOS capacity in pancreatic islets. Breaking the vicious cycle of excess mitochondrial ROS generation and complex II damage increase the expression of NKX6.1, which is involved in pancreatic beta-cell proliferation and maturation, and improve glucose metabolism in the TCA cycle, resulting in the protection of pancreatic beta-cells. Our results would imply that the protection of the mitochondrial ETC complex II in pancreatic beta-cells represents a new therapeutic target for type 2 diabetes.

## Materials and methods

### Animals

Six-week-old male BKS.Cg-Dock7^m^ + / + Lepr^db^/J (*db/db*) mice were purchased from the Oriental Yeast Co. (Tokyo, Japan) and allowed free access to food and water. The room temperature was maintained at 25 °C. Two to three mice were kept in each cage, under controlled conditions, on a 12-h light/dark cycle. The mice were allocated to two groups and fed either standard chow (CE-2, CLEA Japan, Tokyo, Japan) (control group) or standard chow (CE-2) containing 0.01% luseogliflozin (Taisho Pharmaceutical, Tokyo, Japan) (luseo group) from 6 to 10 weeks. We confirmed that the luseogliflozin treatment reduced the blood glucose concentrations of the *db/db* mice (Supplementary Fig. S6). Body weight and food intake were shown in our previous study^11^. All the experimental procedures were approved by the Animal Use Committee of Hokkaido University Graduate School of Medicine (18-0033) and were conducted in compliance with the Animal Use Guidelines of Hokkaido University and the ARRIVE guidelines.

### Islet isolation

Pancreatic islets were isolated from the mice as described previously with minor modifications^42^. Briefly, 2.5 mL Hanks’ balanced salt solution (HBSS) (Sigma-Aldrich, St. Louis, MO) containing 0.9 mg/mL collagenase from *Clostridium histolyticum* (Sigma-Aldrich, St Louis, MO) was injected into the common bile duct after clamping the ampulla of Vater. The swollen pancreas was removed and incubated at 37 °C for 24 min. Digested pancreas was dispersed by pipetting and rinsed with HBSS (Sigma-Aldrich, St. Louis, MO) and fetal bovine serum (Gibco BRL, Paisley, UK). The islets were picked under a microscope using a pipette.

### Microarray analysis

RNA was extracted from isolated islets (four mice per group) using an RNeasy Mini kit (Qiagen, Hilden, Germany). Amplification and hybridization were performed using an Affymetrix GeneChip® Mouse Gene 2.0 ST Array (Affymetrix, Santa Clara, CA). The screening conditions for differentially expressed genes were defined as genes that showed at least a 1.5-fold change.

### Real-time quantitative PCR

RNA was extracted from isolated islets using an RNeasy Mini kit (Qiagen) and used as the starting material for cDNA preparation. Quantitative PCR was performed in duplicate using a 7500 Fast Real-time PCR system with Power SYBR Green PCR Master Mix (Applied Biosystems, Foster City, CA). Glyceraldehyde 3-phosphate dehydrogenase (*Gapdh*) was used as the reference gene. The primer sequences are shown in Supplementary Table S1.

### Preparation of permeabilized islets

After the isolation of 500 islets from three to five mice, they were permeabilized by gentle agitation for 20 min in an ice-cold relaxing solution (BIOPS; CaK_2_EGTA 2.77 mmol/L, EGTA 7.23 mmol/L, taurine 20 mmol/L, MgCl_2_ 6.56 mmol/L, ATP 5.77 mmol/L, phosphocreatine 15 mmol/L, dithiothreitol 0.5 mmol/L, 4-morpholineethanesulfonic acid 50 mmol/L, pH 7.1) containing saponin (final concentration, 50 µg/mL), as described previously, with minor modifications^43^. After the permeabilization, the islets were rinsed twice by agitation for 5 min in an ice-cold respiration medium (MiR05; sucrose 110 mmol/L, K-lactobionate 60 mmol/L, EGTA 0.5 mmol/L, 0.1% essentially fatty acid-free bovine serum albumin, MgCl_2_ 3 mmol/L, taurine 20 mmol/L, KH_2_PO_4_ 10 mmol/L, HEPES 20 mmol/L, pH 7.1).

### Mitochondrial respiratory capacity

The mitochondrial respiratory capacity of the permeabilized islets was measured using a high-resolution respirometer (Oxygraph-2 k; Oroboros Instruments, Innsbruck, Austria) at 37 °C, as described previously^43–45^. After the addition of the permeabilized islet suspension (final concentration, 250 islets/mL) to the chamber of the Oxygraph-2 k, we added respiratory substrates, ADP, and inhibitors to measure mitochondrial respiration during each state in the following order: (1) glutamate (final concentration, 5 mmol/L) and malate (1 mmol/L), (2) ADP (1.25 mmol/L), (3) succinate (10 mmol/L), (4) rotenone (0.5 µmol/L), (5) antimycin A (5 µmol/L), (6) ascorbate (0.5 mmol/L), and N,N,N’,N’-tert-methyl-p-phenyldiamine (TMPD, 2 mmol/L), and (7) sodium azide (10 mmol/L).

We evaluated leak state respiration using complex I-linked substrates (glutamate and malate) (CI_L, state 2 [non-ADP stimulated] respiration), oxidative phosphorylation (OXPHOS) capacity (state 3 [ADP-stimulated] respiration) using complex I-linked substrates (CI_P), and OXPHOS capacity using complex I + II-linked substrates (glutamate, malate, and succinate [complex II-linked substrate]) (CI + II_P). Then, complex II-linked OXPHOS capacity (CII_P) was evaluated in the presence of complex I + II-linked substrates using a complex I inhibitor (rotenone). After the addition of a complex I inhibitor and a complex III inhibitor (antimycin A), residual oxygen consumption (ROX), non-mitochondrial oxygen consumption, was evaluated, and this value was subtracted from the oxygen consumption rate during each state in which mitochondrial respiratory capacity was calculated. The capacity of complex IV was also evaluated after the addition of ascorbate and TMPD. Because of the high levels of auto-oxidation of TMPD, a complex IV inhibitor (sodium azide) was added and the difference between the two levels obtained was calculated (the specific complex IV capacity). The oxygen consumption rate is expressed as pmol s^−1^islet^−1^. DatLab software version 7.0 (Oroboros Instruments) was used for data analysis.

The respiratory control ratio (RCR) was calculated as state 3 respiration/state 2 respiration, and the substrate control ratio (SCR) was calculated as state 3 respiration with either complex I-linked substrates or complex II-linked substrates/state 3 respiration with complex I + II-linked substrates.

### Mitochondrial reactive oxygen species generation

We measured mitochondrial ROS generation simultaneously with the mitochondrial respiratory capacity in the permeabilized islets using a spectrofluorometer (Fluorescence LED2-Module; Oroboros Instruments) equipped with a respirometer, as previously described^44,45^. Mitochondrial ROS concentration was evaluated after the conversion of mitochondrial superoxide (O_2_^−^) into hydrogen peroxide (H_2_O_2_) by adding superoxide dismutase (SOD). After the addition of permeabilized islets to the chamber of the Oxygraph-2 k, we subjected SOD (5 U/mL), horseradish peroxidase (1 U/mL), and Amplex® UltraRed reagent (10 µmol/L; Thermo Fisher Scientific, Waltham, MA). H_2_O_2_ reacts with Amplex UltraRed in 1:1 stoichiometry, the reaction is catalyzed by horseradish peroxidase, and it yields the fluorescent compound resorufin. The excitation wavelength was 525 nm, and fluorescence detection was at 587 nm. The fluorescence of resorufin was continuously monitored during the measurements of mitochondrial respiratory capacity. The H_2_O_2_ generation rate was calibrated by titrating H_2_O_2_ in 0.1 µmol/L increments before and after the addition of each substrate, to eliminate the possible interference of substrates. The H_2_O_2_ generation rate was expressed as pmol s^−1^islet^−1^.

### Immunohistochemistry

Isolated pancreata were immersion-fixed in 4% paraformaldehyde at 4 °C overnight, then paraffin-embedded, and 5-µm sections were mounted on glass slides. For immunofluorescence, tissue sections were incubated at 4 °C overnight with the primary antibodies listed in Supplementary Table S2. After rinsing with PBS, the sections were incubated with secondary antibodies for 30 min. Immunofluorescence images were acquired using a BZ-II analyzer (Keyence, Osaka, Japan). The mitochondrial area was quantified using a BZ-II analyzer (Keyence, Osaka, Japan). The mitochondrial area/beta-cells ratio was expressed as the Tom 20 positive area inside the insulin positive area per islet divided by the number of beta-cells. The Nkx6.1 positive cells/beta-cells ratio was expressed as the number of cells with strong nucleic Nkx6.1 staining among the insulin positive cells per islet divided by the number of beta-cells.

### Electron microscopy

Isolated pancreatic tissue was fixed in 2.5% glutaraldehyde in 0.1 mmol/L phosphate buffer (pH 7.2) for 3 h at 4 °C and post-fixed in 1% osmium tetroxide in 0.1 mmol/L phosphate buffer (pH 7.2) for 90 min at 4 °C. It was then serially dehydrated in ethanol and embedded in epoxy resin. Sections were cut on an LKB ultramicrotome, and consecutive ultrathin sections were mounted on copper grids and stained with 3% uranyl acetate and 0.2% lead citrate. They were then examined using a transmission electron microscope (H-7,100; Hitachi, Tokyo, Japan).

### Metabolomic analysis

Isolated islets were incubated as previously described^46^, followed by stored at −80 °C. Five to 10 mg of frozen isolated islets (250 islets/sample isolated from two to three mice) were placed into 225 µL of 50% acetonitrile/Milli-Q water containing internal standards (H3304-1002, Human Metabolome Technologies, Inc., Tsuruoka, Japan) at 0 °C to inactivate enzymes. The tissue was homogenized at 1500 rpm for 120 s using a tissue homogenizer (Micro Smash MS100R, Tomy Digital Biology Co., Ltd., Tokyo, Japan) and the homogenate was centrifuged at 2300×*g* and 4 °C for 5 min. Subsequently, 400 µL of the upper aqueous layer was centrifugally filtered through a Millipore 5-kDa cut-off filter at 9100×*g* and 4 °C for 120 min to remove proteins. The filtrate was centrifugally concentrated and re-suspended in 25 µL of Milli-Q water for capillary electrophoresis-mass spectrometry analysis. The measurements were corrected by the tissue weight. Metabolomic analysis was performed at Human Metabolome Technologies, Inc., Tsuruoka, Japan.

### Statistical analyses

Data are expressed as mean ± standard deviation (SD). Student’s *t*-test was used for comparisons between two groups. In the pathway analysis, two-tailed Fisher’s exact tests were used for comparisons between two groups. *P* < 0.05 was considered to represent statistical significance. Statistical analyses were performed using JMP Pro version 14 (SAS Inc., Cary, NC).

## Supplementary Information

Supplementary Information.

## Data Availability

All the data from this article are available from the corresponding author upon request.
