# Supplementary material for: Luseogliflozin preserves the pancreatic beta-cell mass and function in db/db mice by improving mitochondrial function
Source: Sci Rep. 2022 Jun 13;12:9740. doi: 10.1038/s41598-022-13888-6 (PMC9192642; doi:10.1038/s41598-022-13888-6)
Supplement: Supplementary file 1 — Supplementary Information. [file 41598_2022_13888_MOESM1_ESM.pdf]

SUPPLEMENTAL MATERIAL

Luseogliflozin preserves the pancreatic beta-cell mass and function in *db/db* mice by improving mitochondrial function

Yuki Yamauchi [1], Akinobu Nakamura [1,*], Takashi Yokota [2,3], Kiyohiko Takahashi [1], Shinichiro Kawata [1], Kazuhisa Tsuchida [1], Kazuno Omori [1], Hiroshi Nomoto [1], Hiraku Kameda [1], Kyu Yong Cho [1,2], Toshihisa Anzai [3], Shinya Tanaka [4,5], Yasuo Terauchi [6], Hideaki Miyoshi [1,7], Tatsuya Atsumi [1]

[1] Department of Rheumatology, Endocrinology and Nephrology, Faculty of Medicine and Graduate School of Medicine, Hokkaido University, Sapporo, Japan

[2] Clinical Research and Medical Innovation Center, Hokkaido University Hospital, Sapporo, Japan

[3] Department of Cardiovascular Medicine, Faculty of Medicine and Graduate School of Medicine, Hokkaido University, Sapporo, Japan

[4] Department of Cancer Pathology, Faculty of Medicine, Hokkaido University, Sapporo, Japan

[5] Institute for Chemical Reaction Design and Discovery (WPI-ICReDD), Hokkaido University, Sapporo, Japan

[6] Department of Endocrinology and Metabolism, Graduate School of Medicine, Yokohama City University, Yokohama, Japan

[7] Division of Diabetes and Obesity, Faculty of Medicine and Graduate School of Medicine Hokkaido University, Sapporo, Japan

***Corresponding author**: Akinobu Nakamura

Department of Rheumatology, Endocrinology and Nephrology, Faculty of Medicine and Graduate School of Medicine, Hokkaido University, Kita-15 Nishi-7, Kita-Ku, Sapporo 060-8638, Japan.

Tel: +81-11-706-5915; Fax: +81-11-706-7710; E-mail: akinbo@tim.hi-ho.ne.jp

Supplementary Methods.

Western Blot Analysis

To prepare whole-cell protein extracts, frozen pancreatic islets were homogenized in lysis buffer (150 mM HEPES (pH 7.0), 250 mM NaCl, 0.1% Nonidet P-40, 5 mM EDTA, 50 mM sodium fluoride, 0.2 mM sodium orthovanadate) with protease and phosphatase inhibitors (0.1% leupeptin, 0.1% aprotinin, 0.5 mM dithiothreitol, 1 mM phenylmethylsulfonyl fluoride). Islets extracts were centrifuged at 15,000 rpm at 4 °C for 10 min. The supernatant was collected for use in western blotting. Proteins were separated by SDS-PAGE (12%) and transferred onto polyvinylidene difluoride membranes (Bio-Rad Laboratories, Inc. Hercules, CA). Membranes were blocked with Bullet Blocking One for Western Blotting (Nacalai Tesque, Kyoto, Japan) for 30 min. After blocking, membranes were cut prior to hybridisation with antibodies and were probed overnight at 4 °C with Total OXYPHOS antibody (Abcam 110413, Cambridge, UK, 1:500) and β-actin antibody (Santa Cruz Biotechnology sc-1615, Dallas, TX, 1:500). Protein bands were visualized using ECL reagents (Global Life Sciences Solutions Operations, Amersham, UK), and images were obtained using a LAS-4000 UV mini CCD camera system (Fujifilm Co., Tokyo, Japan).

Cell culture

The rat insulinoma cell line INS 832/13 was purchased from Sigma-Aldrich, St. Louis, MO. INS 832/13 cells were cultured in RPMI 1640 medium (11 mM glucose) supplemented with 10mM HEPES, 1 mM sodium pyruvate, 100 IU/mL penicillin and 100 mg/mL streptomycin, 10% heat-inactivated fetal bovine serum (FBS) (Gibco BRL, Paisley, UK) at 37 °C in a humidified 5% CO_2 atmosphere. INS 832/13 cells were used between passages 8 and 11 and seeded at a density of 0.6×10^6 cells/well in 6-well plates. The cells were treated with DMSO vehicle (Luseo-) or vehicle + 100 nM luseogliflozin (Luseo+) in RPMI 1640 medium containing 11 mM or 22 mM glucose for 48 h.

Supplementary Table S1. The primer sequences for real-time quantitative PCR

Primer name	Sequence
Gapdh	
Forward	GGCCCCTCTGGAAAGCTGTGGTGT
Reverse	GTTGGGGGCCGAGTTGGGATAGG
Slc2a2	
Forward	TGTGCTGCTGGATAAATTCG
Reverse	TTCAGCAACCATGAACCAAG
Pcx	
Forward	CTGAAGTTCCAAACAGTTCGAGG
Reverse	CGCACGAAACACTCGGATG
Cs	
Forward	AACTCAGGACGGGTTGTTCCAG
Reverse	TAGTAATTCATCTCCGTCATGCC
Aco2	
Forward	TGGGTGGTGATTGGAGATGA
Reverse	ATCTGGGTCTCGTTGAAGGT
Idh2	
Forward	GAAGGTGTGCGTGGAGAC
Reverse	CCGTGGTGTTCAGGAAGT
Ogdh	
Forward	TGCAGATGTGCAATGATGAC
Reverse	GCAGCACATGGAAGAAGTTG
Sdha	
Forward	GGAACACTCCAAAAACAGACCT
Reverse	CCACCACTGGGTATTGAGTAGAA
Mdh2	
Forward	TTCAACACCAACGCTACCATTGTG
Reverse	GTGTTCGCTCTGACGATGTCAAGG
Nkx6.1	
Forward	CTGCACAGTATGGCCGAGATG
Reverse	CCGGGTTATGTGAGCCCAA
Ccnd2	
Forward	AAGCCTGCCAGGAGCAAA
Reverse	ATCCGGCGTTATGCTGCTCT
Drp1	
Forward	TAAGCCCTGAGCCAATCCATC
Reverse	CATTCCCGGTAAATCCACAAGT
Mfn1	
Forward	CCTACTGCTCCTTCTAACCCA
Reverse	AGGGACGCCAATCCTGTGA

Supplementary Table S2. The antibodies used for immunofluorescence.

Antigen	Source	Dilution	Company, Catalog#	RRID
**Primary antibodies**				
Insulin	Guinea pig	1:1	Dako, IR002	AB_2800361
Tom20	Rabbit	1:800	Cell Signaling Technology, 42406	AB_2687663
Nkx6.1	Rabbit	1:1500	Cell Signaling Technology, 54551	AB_2722625
Glucagon	Mouse	1:200	Sigma, G2654	AB_259852
Drp1	Rabbit	1:1000	Abcam, ab184247	AB_2895215
Mfn1	Rabbit	1:100	Proteintech, 13798-1-AP	AB_2266318
**Secondary antibodies**				
Guinea pig IgG (Alexa Fluor 488)	Goat	1:200	Life Technologies, A11073	AB_2534117
Rabbit IgG (Alexa Fluor 594)	Goat	1:200	Life Technologies, A11012	AB_2534079
Mouse IgG (Cyanine 5)	Goat	1:200	Life Technologies, A10524	AB_2534033

Supplementary Figure S1. Effects of luseogliflozin on gene expressions of *Cox6a2, Nkx6.1,* and *Ccnd2* in pancreatic islets of 10-week-old *db/db* mice

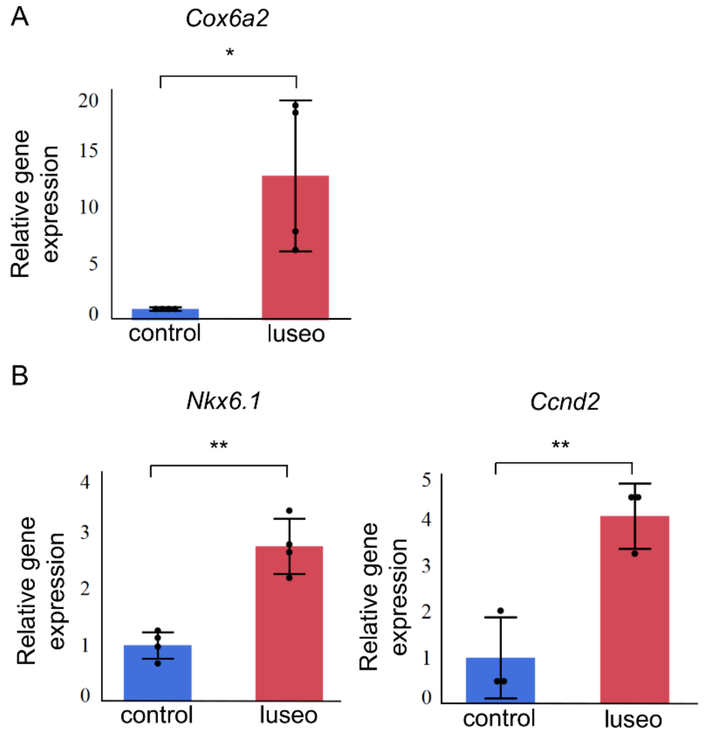

A. Gene expressions of *Cox6a2* in the control group and the luseo group by real-time PCR (n = 4). **B.** Gene expressions of *Nkx6.1* and *Ccnd2* in the control group and the luseo group by real-time PCR (control group n = 3, luseo group n = 4). The data has been normalized by GAPDH expression. Values are mean ± SD. *P* values were determined using Student's *t* test. * $P < 0.05$; ** $P < 0.01$. *Cox6a2*, Cytochrome c oxidase subunit 6A2; *Nkx6.1*, NK6 homeobox 1; *Ccnd2*, Cyclin D2.

Supplementary Figure S2. Effects of luseogliflozin on mitochondrial respiratory capacity in pancreatic islets of 10-week-old *db/db* mice

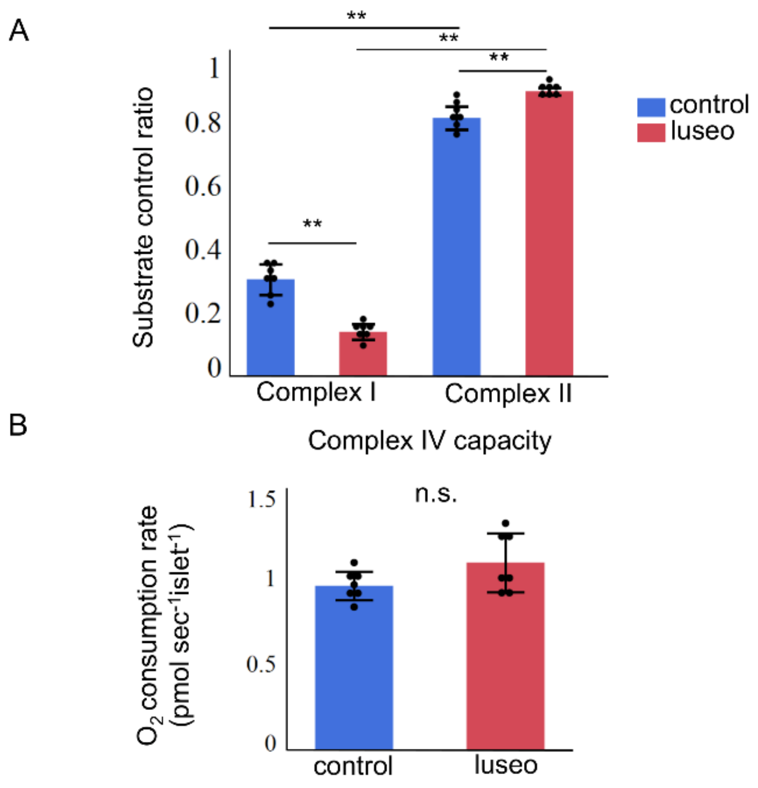

A. Substrate control ratio in the control group and the luseo group. Complex I substrate control ratio was the ratio complex I-linked oxidative phosphorylation to complex I+II-linked oxidative phosphorylation (n = 7). Complex II substrate control ratio was the ratio complex II-linked oxidative phosphorylation to complex I+II-linked oxidative phosphorylation (n = 7). **B.** Complex IV respiratory capacity in the control group and the luseo group (n = 7). Values are mean ± SD. *P* values were determined using Student's *t* test. ** *P* < 0.01. n.s.; not significant.

Supplementary Figure S3. Effects of luseogliflozin on gene expression of *Nkx6.1* in INS-1 832/13 cells.

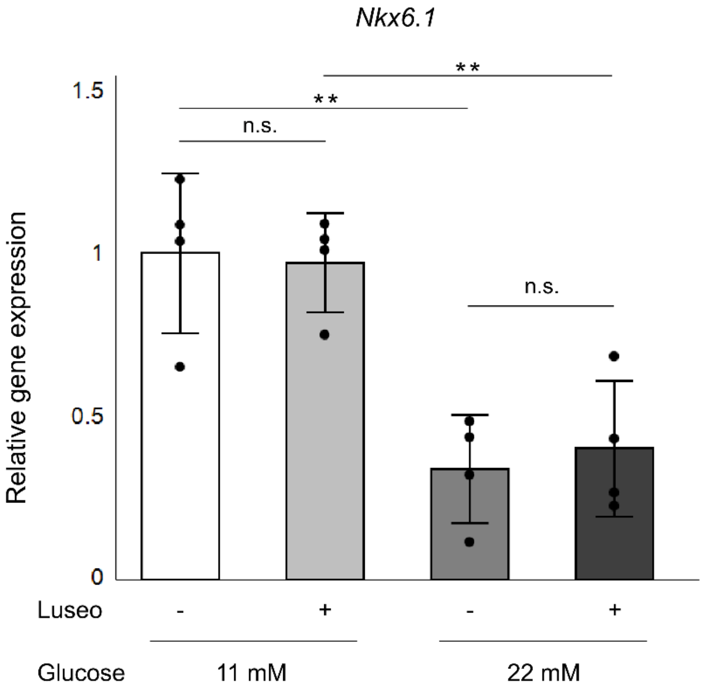

Gene expression of *Nkx6.1* in INS 832/13 cells treated with DMSO vehicle (Luseo-) or vehicle + 100 nM luseogliflozin (Luseo+) in RPMI 1640 medium containing 11 mM or 22 mM glucose for 48 h (n = 4). Values are mean ± SD. *P* values were determined using Tukey's HSD test. ** $P < 0.01$. n.s.; not significant.

Supplementary Figure S4. Effects of luseogliflozin on protein expressions of mitochondrial complexes in pancreatic islets of 10-week-old *db/db* mice.

A

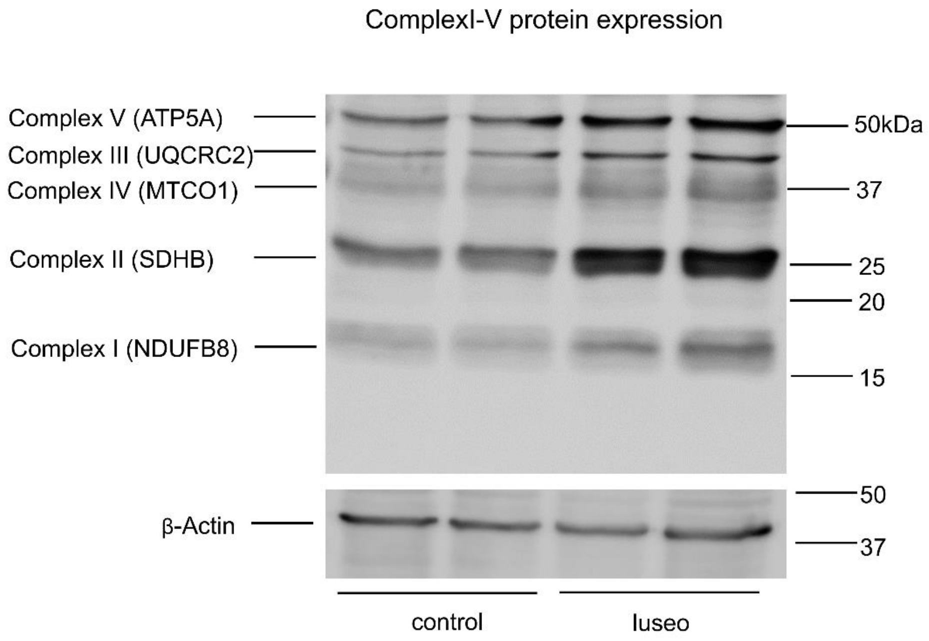

B

Complex I-V (uncropped image)

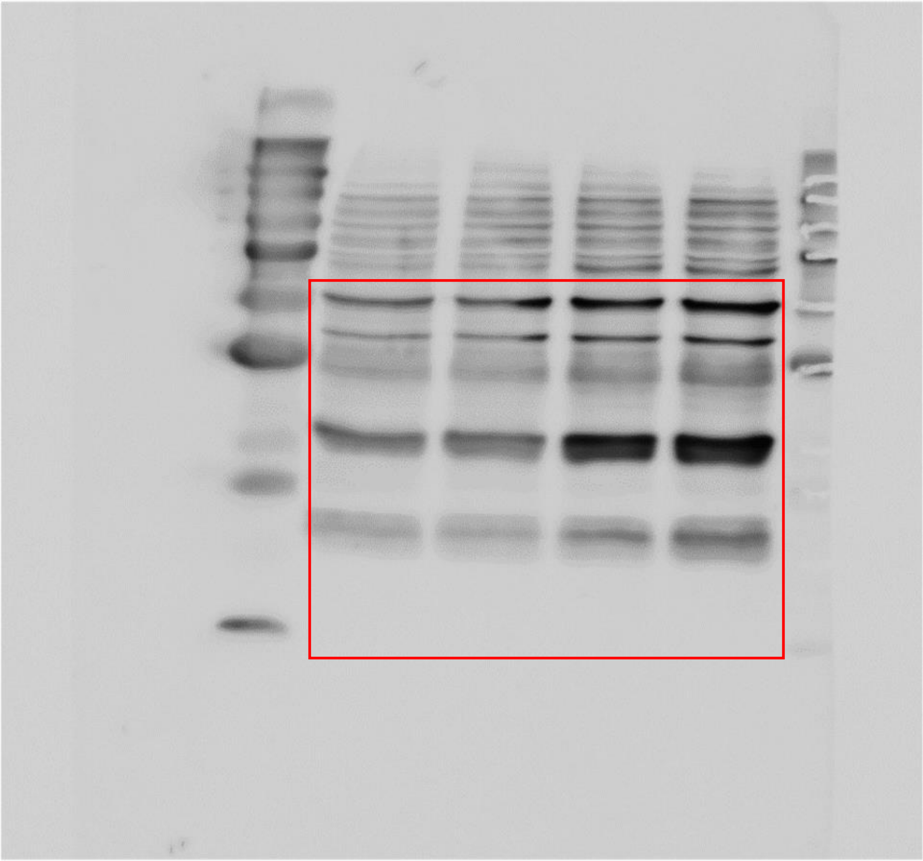

C

β-actin (uncropped image)

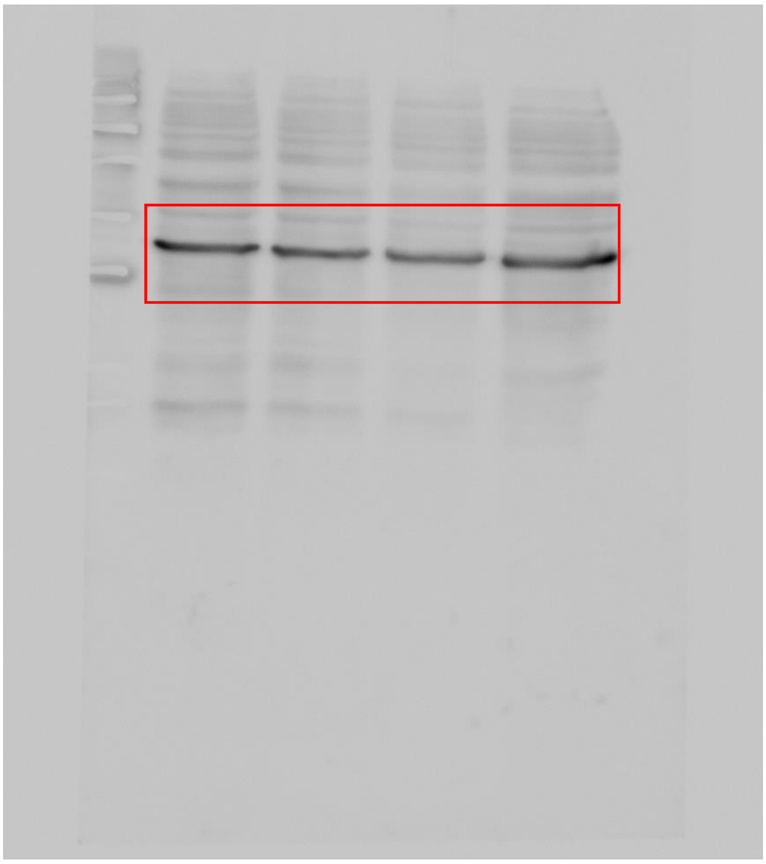

A. Protein levels of representative subunits from complex I-V in the control group and luseo group (n = 2, 15 μg/lane). β-actin was used as the loading control. **B.** The uncropped image for Complex I-V protein. **C.** The uncropped image for β-actin. Boxes indicate the cropped regions.

Supplementary Figure S5. Effects of luseogliflozin on Drp1 and Mfn1 expression in pancreatic beta-cells of 10-week-old *db/db* mice.

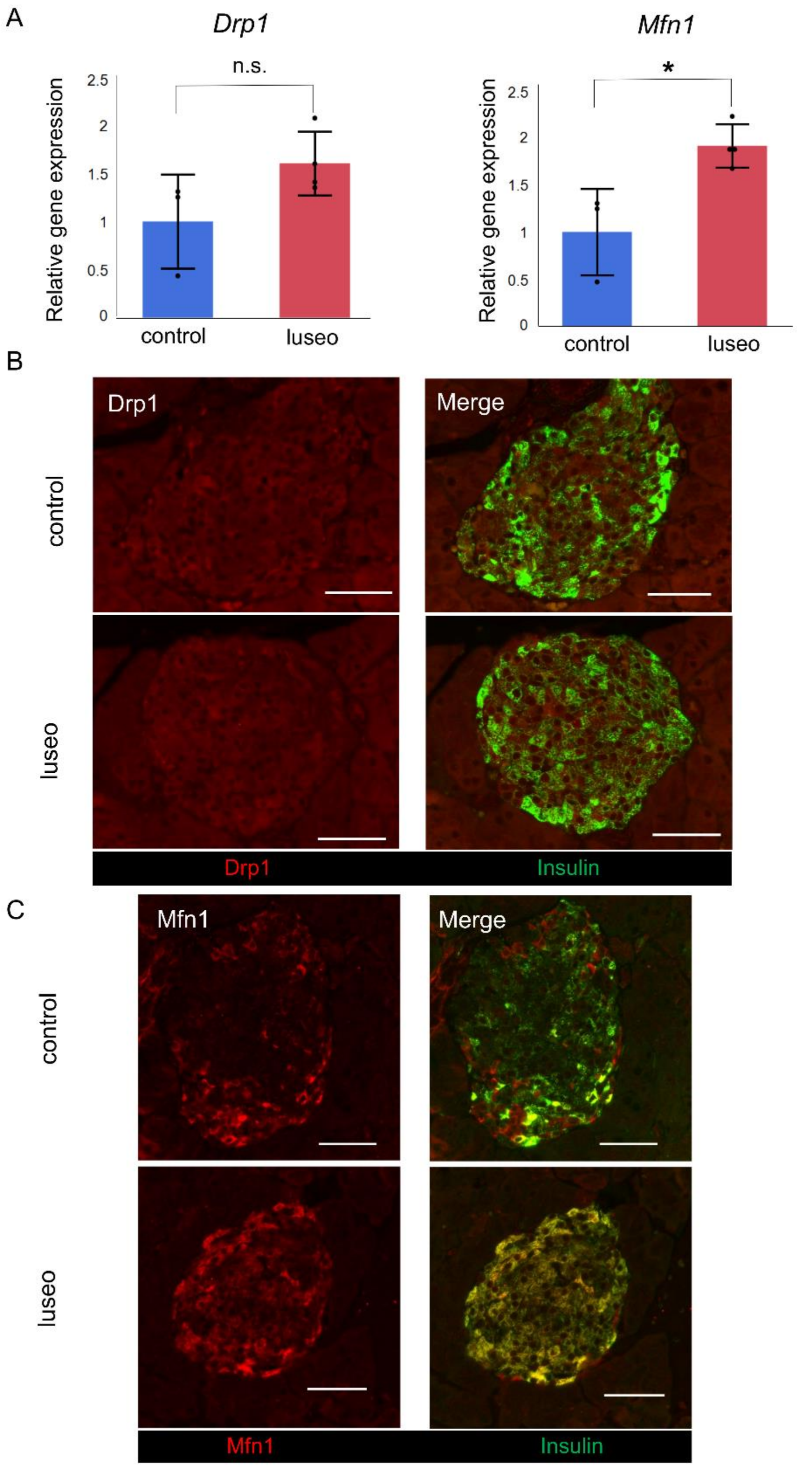

A. Gene expression of *Drp1* and *Mfn1* in the control group and luseo group by real-time PCR (control group n = 3, luseo group n = 4). The data has been normalized by GAPDH expression. Values are mean ± SD. *P* values were determined using Student's *t* test. * $P < 0.05$. n.s.; not significant. **B.** Images of pancreatic beta-cells from the control and luseo groups stained for Drp1 (red) and insulin (green). Scale bars: 50 μm. **C.** Images of pancreatic beta-cells from the control and luseo groups stained for Mfn1 (red) and insulin (green). Scale bars: 50 μm.

Supplementary Figure S6. Effects of luseogliflozin on blood glucose levels in *db/db* mice

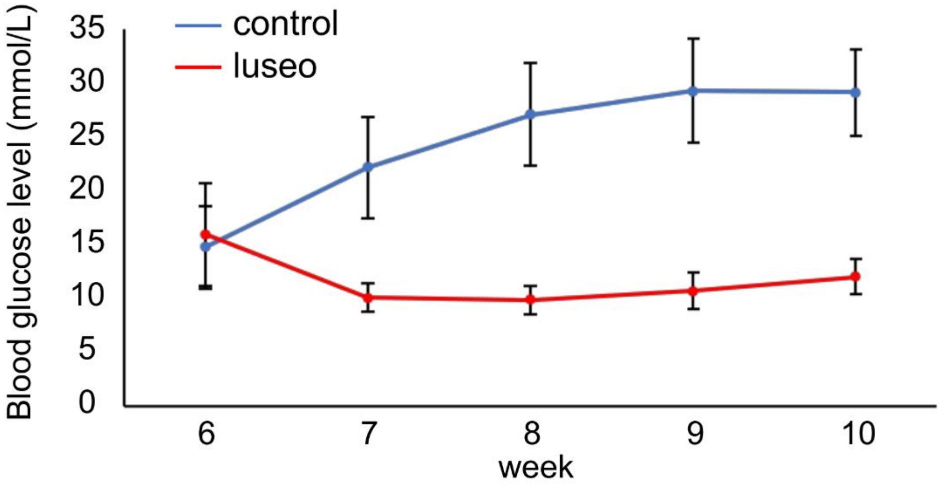

Blood glucose levels in the control group and the luseo group for 4 weeks (n=30). Values are mean ± SD.
